# Achieving postprandial glucose control with lixisenatide improves glycemic control in patients with type 2 diabetes on basal insulin: a post-hoc analysis of pooled data

**DOI:** 10.1186/s40842-019-0088-5

**Published:** 2020-01-14

**Authors:** Jaime A. Davidson, William Stager, Sachin Paranjape, Rachele Berria, Lawrence A. Leiter

**Affiliations:** 1 Department of Internal Medicine, Touchstone Diabetes Center, The University of Texas Southwestern Medical Center, 5323 Harry Hines Boulevard, K5.246, Dallas, TX 75390-8549 USA; 2 Sanofi US, Inc, Bridgewater, NJ USA; 3 Li Ka Shing Knowledge Institute, St Michael’s Hospital, University of Toronto, Toronto, ON Canada

**Keywords:** Type 2 diabetes, Lixisenatide, Glucagon-like peptide-1 receptor, Post-prandial glucose, Glycemic targets

## Abstract

**Background:**

To examine the impact on glycemic control of achieving postprandial glucose (PPG) target with lixisenatide, a once-daily glucagon-like peptide-1 receptor agonist approved in the US, in patients with uncontrolled type 2 diabetes (T2D) on basal insulin, an agent that primarily targets fasting plasma glucose.

**Methods:**

A post hoc pooled analysis was conducted using clinical trial data extracted from the intent-to-treat subpopulation of patients with T2D who participated in the 24-week, phase 3, randomized, double-blind, placebo-controlled, 2-arm parallel-group, multicenter GetGoal-L (NCT00715624), GetGoal-Duo 1 (NCT00975286) and GetGoal-L Asia trials (NCT00866658).

**Results:**

Data from 587 lixisenatide-treated patients and 484 placebo-treated patients were included. Patients on lixisenatide were more likely to achieve a PPG target of < 10 mmol/L (< 180 mg/dL) than placebo-treated patients (P < 0.001), regardless of baseline fasting plasma glucose (FPG) levels. More importantly, those who reached the PPG target experienced a significantly greater reduction in mean HbA1c, were more likely to achieve HbA1c target of < 53 mmol/mol (< 7.0%), and experienced weight loss. Those outcomes were achieved with no significant differences in the risk of symptomatic hypoglycemia compared with placebo.

**Conclusion:**

Compared with placebo, addition of lixisenatide to basal insulin improved HbA1c and reduced PPG, without increasing hypoglycemia risk. These findings highlight the importance of PPG control in the management of T2D, and provide evidence that adding an agent to basal insulin therapy that also impacts PPG has therapeutic value for patients who are not meeting glycemic targets.

**Trial registration:**

NCT00715624. Registered 15 July 2008, NCT00975286*.* Registered 11 September 2009, NCT00866658*.* Registered 20 March 2009.

## Background

Controlling hyperglycemia in patients with type 2 diabetes (T2D), as measured by glycated hemoglobin (HbA1c), is essential to reduce long-term risks of diabetes-related microvascular damage to kidneys, nerves, and eyes, as well as other cardiovascular complications [1–3]. The American Diabetes Association (ADA) recommends target HbA1c levels of < 48 mmol/mol (< 6.5%), < 53 mmol/mol (< 7.0%), or < 64 mmol/mol (< 8.0%), tailored to the needs of the patient [4], and the International Diabetes Federation (IDF) and the American Association of Clinical Endocrinologists (AACE)/American College of Endocrinology (ACE) advise a target HbA1c level of < 48 mmol/mol (< 6.5%), while also emphasizing individualized goal-setting [5, 6].

Due to the progressive nature of T2D, many patients eventually require the use of basal insulin to reach and maintain glycemic targets [4, 7]. However, many patients and health care providers fail to intensify treatment in a timely fashion [8]. The HbA1c level reflects contributions from both fasting plasma glucose (FPG) and postprandial plasma glucose (PPG). PPG has been shown to play a predominant role in residual hyperglycemia as HbA1c levels approach 53 mmol/mol (7.0%) and FPG levels are within target range (4.4–7.2 mmol/L [80–130 mg/dL]) [4, 9]. In patients with T2D and uncontrolled hyperglycemia on OADs, treatment intensification with basal insulin resulted in HbA1c and FPG reductions, while PPG accounted for approximately two-thirds of residual hyperglycemia, suggesting an important role for PPG-targeting therapies in helping patients to achieve glycemic goals [10].

Lixisenatide is a once-daily GLP-1 RA that lowers PPG, reduces appetite, and leads to weight loss, which is typical of the GLP-1 RA class [11–13]. Like other GLP-1 RAs, lixisenatide is associated with a very low risk of hypoglycemia due to its glucose-dependent mechanism of action [6, 12, 13]. While also impacting FPG, treatment of patients with T2D with lixisenatide results in robust reductions in PPG [14]. The pronounced effects of lixisenatide on lowering PPG provide a rationale for combining lixisenatide with basal insulin to achieve additive effects on glycemic control [15].

Because of the recognized contribution of PPG to overall hyperglycemia and the impact of lixisenatide on PPG, it was hypothesized that reducing PPG to < 10 mmol/L (< 180 mg/dL), as recommended by the ADA, would also increase the likelihood of patients with T2D achieving HbA1c < 53 mmol/mol (< 7.0%). In this study, the contribution of lixisenatide to achievement of ADA-recommended PPG target was investigated in patients with T2D uncontrolled on basal insulin. In addition, we evaluated whether achieving PPG targets affected HbA1c and other efficacy and safety outcomes.

## Methods

### Study design

This post hoc pooled analysis used clinical trial data extracted from the intent-to-treat subpopulation of patients with T2D who participated in standardized meal tests (measured 2 h after a standard liquid breakfast) as part of the 24-week, phase 3, randomized, double-blind, placebo-controlled, 2-arm parallel-group, multicenter GetGoal-L (NCT00715624) [16], GetGoal-Duo1 (NCT00975286) [17], and GetGoal-L Asia (NCT00866658) [18] trials; all trials are registered at ClinicalTrials.gov. These were the 3 trials that evaluated the efficacy and safety of adding lixisenatide to basal insulin therapy in patients with T2D inadequately controlled on basal insulin, with or without OADs (metformin [MET], thiazolidinediones [TZD], or sulfonylurea [SU]). GetGoal-L enrolled patients from 15 countries who were inadequately controlled (HbA1c = 53–86 mmol/mol [7.0–10.0%]) on an existing, stable dose of basal insulin therapy for ≥3 months with or without MET [16]. Patients in GetGoal-L Asia were from Japan, Republic of Korea, Taiwan, and the Philippines. These patients were on existing basal insulin therapy with or without a SU. Patients in GetGoal-Duo 1 were from 25 countries and were inadequately controlled (HbA1c 53–86 mmol/mol [7.0–10.0%]) on existing OAD therapy. If present, SU therapy was discontinued, and patients were initiated on basal insulin therapy with or without MET or a TZD during the run-in phase. After the 12-week run in, patients with an HbA1c ≥ 53 mmol/mol (≥ 7.0%) to ≤75 mmol/mol (≤ 9.0%) and FPG ≤ 7.8 mmol/L (≤ 140 mg/dL) were randomly assigned to add lixisenatide or placebo [17].

To evaluate the impact of baseline FPG on achieving glycemic control, patients with baseline FPG of < 7 mmol/L (< 126 mg/dL) were categorized as controlled, whereas those with FPG ≥ 7 mmol/L (≥ 126 mg/dL) were categorized as uncontrolled. Patients were further grouped into PPG responders (< 10 mmol/L [< 180 mg/dL]) or non-responders (≥ 10 mmol/L [≥ 180 mg/dL]) at the end of the 24-week treatment period.

### Assessments

The primary endpoint was the proportion of patients who achieved PPG < 10 mmol/L (< 180 mg/dL) at Week 24. Secondary endpoints in PPG responders and non-responders with or without controlled FPG levels at baseline were change in mean HbA1c from baseline to Week 24; the percentage of patients with HbA1c < 53 mmol/mol (< 7.0%) at Week 24; body weight change over 24 weeks; and the rate of symptomatic hypoglycemia during the study period (defined as typical symptoms of hypoglycemia accompanied by an SMPG value of ≤60 mg/dL [3.9 mmol/L]). Hypoglycemia was recorded via patient diaries; patients recorded any hypoglycemic events daily, which were passed on to investigators at the next visit.

### Statistical analyses

Continuous efficacy assessments were analyzed using analysis of variance carried out by baseline FPG category using last observation carried forward at Week 24, with treatment group and PPG category as fixed effects. Response rates and hypoglycemia rates were analyzed using chi-square tests. Analyses excluded measurements obtained after the use of rescue medication and/or after treatment cessation. Rescue medication of short-acting or rapid-acting insulin was given to patients with a measurement of FPG > 11.1 mmol/L (> 200 mg/dL) or > 75 mmol/mol (HbA1c > 9%), for 3 consecutive days (Weeks 0–8), when changes to diet and study medication did not resolve the high readings. This threshold changed to FPG > 10 mmol/L (> 180 mg/dL) or HbA1c > 69 mmol/mol (> 8.5%) for Weeks 8–24. Results were combined across studies using a fixed-effects meta-analysis with inverse variance weights calculated separately by FPG and PPG categories. All analyses were performed using SAS Version 9.2® [SAS Institute Inc. Cary, NC, USA] or higher.

## Results

### Patient baseline characteristics

Data from 587 lixisenatide-treated patients and 484 placebo-treated patients were included in this analysis. Baseline characteristics between the 2 treatment groups were comparable. Females made up 53% of patients in the lixisenatide group and 50% in the placebo group. In the lixisenatide versus placebo groups, respectively, mean body weight was 82 kg versus 81 kg, and mean duration of T2D was 11.8 years versus 11.3 years. Mean HbA1c was 65 mmol/mol (8.1%) in both groups.

When baseline variables were examined in the groups stratified by baseline FPG and PPG goal achievement the majority of variables were similar between the groups (Table 1). In the group with controlled FPG, the placebo PPG responders were less likely to be female and had a lower baseline HbA1c (Table 1). Baseline HbA1c was higher in lixisenatide non-responders with uncontrolled FPG than in responders. Both groups of non-responders with controlled FPG at baseline had higher baseline PPG, though there was no differences between the groups with uncontrolled FPG at baseline.

**Table 1:** Baseline demographic and clinical characteristics by baseline-FPG and PPG-subgroup

	Baseline FPG < 7 mmol/L (< 126 mg/dL) (controlled)				Baseline FPG ≥ 7 mmol/L (≥ 126 mg/dL) (uncontrolled)			
	PPG responders^a^ Lixisenatide (*n* = 156)	PPG non-responders^b^ Lixisenatide (*n* = 127)	PPG responders^a^ Placebo (*n* = 47)	PPG non-responders^b^ Placebo (*n* = 210)	PPG responders^a^ Lixisenatide (*n* = 147)	PPG non-responders^b^ Lixisenatide (*n* = 157)	PPG responders^a^ Placebo (*n* = 23)	PPG non-responders^b^ Placebo (*n* = 204)
Female, n (%)	88 (56.4)	66 (52.0)	18 (38.3)	119 (56.7)	72 (49.0)	84 (53.5)	9 (39.1)	96 (47.0)
Mean age, years (SD)	58.1 (9.6)	58.9 (9.6)	56.0 (9.6)	57.6 (9.8)	55.5 (10.2)	56.7 (10.0)	56.7 (9.7)	56.4 (10.1)
Mean duration of diabetes, years (SD)	12.6 (7.3)	13.6 (7.1)	9.0 (7.0)	11.8 (7.3)	10.5 (6.6)	10.8 (6.5)	11.7 (6.0)	11.2 (6.8)
Mean BMI, kg/m^2^ (SD)	29.8 (6.3)	30.9 (6.4)	31.1 (6.8)	29.4 (6.5)	29.9 (6.3)	31.1 (6.4)	30.8 (5.9)	29.8 (6.5)
Mean HbA1c, mmol/mol (SD)	62 (8.1)	63 (8.3)	57 (6.4)	63 (8.1)	67 (9.1)	69 (9.6)	66 (8.5)	68 (9.0)
Mean FPG, mmol/L (SD)	5.50 (0.9)	5.52 (0.9)	5.57 (0.9)	5.65 (0.9)	8.94 (1.9)	9.27 (2.0)	9.23 (1.7)	9.28 (1.8)
Mean PPG, mmol/L (SD)	14.08 (4.1)	15.24 (3.9)	11.58 (3.8)	14.65 (4.2)	16.21 (4.3)	16.75 (4.4)	14.09 (3.8)	16.67 (4.2)
Mean insulin dose. U (SD)	42.4 (35.9)	45.2 (24.4)	40.3 (14.7)	41.2 (24.9)	41.9 (27.6)	43.0 (24.1)	53.0 (27.1)	41.1 (29.0)

Statistics were derived from pooled data

*Abbreviations: HbA1c* glycated hemoglobin, *BMI* body mass index, *FPG* fasting plasma glucose, *PPG* postprandial glucose, *SD* standard deviation

^a^PPG < 10 mmol/L (< 180 mg/dL); ^b^PPG ≥ 10 mmol/L (≥ 180 mg/dL)

### Impact of lixisenatide treatment on PPG target

**Fig. 1:**
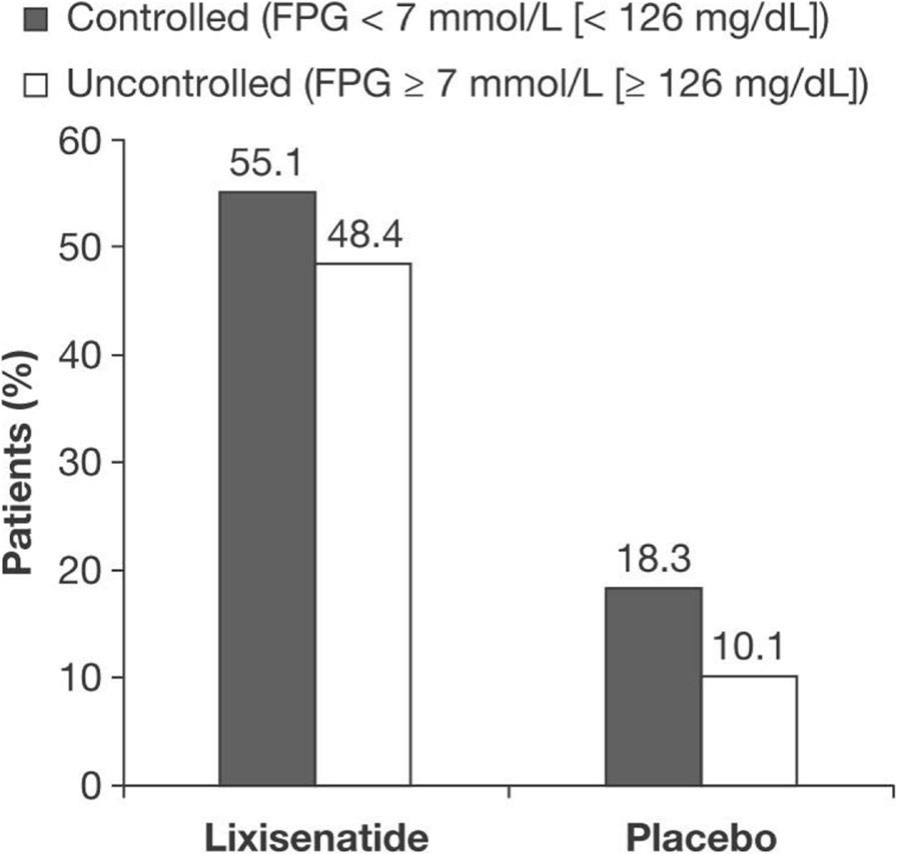
Percentage of patients who achieved ADA-recommended PPG target < 10 mmol/L (< 180 mg/dL) at Week 24. *Abbreviations: ADA* American Diabetes Association, *FPG* fasting plasma glucose, *PPG* postprandial glucose

Significantly more patients who received lixisenatide than those who received placebo achieved the PPG target. In patients with controlled FPG at baseline, 55.1% receiving lixisenatide achieved PPG < 10 mmol/L (< 180 mg/dL), compared with 18.3% in the patients receiving placebo, a difference of 36.8% (*P* < 0.001) (Fig. 1). Similarly, for patients with uncontrolled FPG at baseline, 48.4% in the lixisenatide group, compared with 10.1% in the placebo group, achieved PPG < 10 mmol/L (< 180 mg/dL), a difference of 38.3% (*P* < 0.001) (Fig. 1).

### Impact of achieving PPG target on other efficacy and safety outcomes

**Fig. 2:**
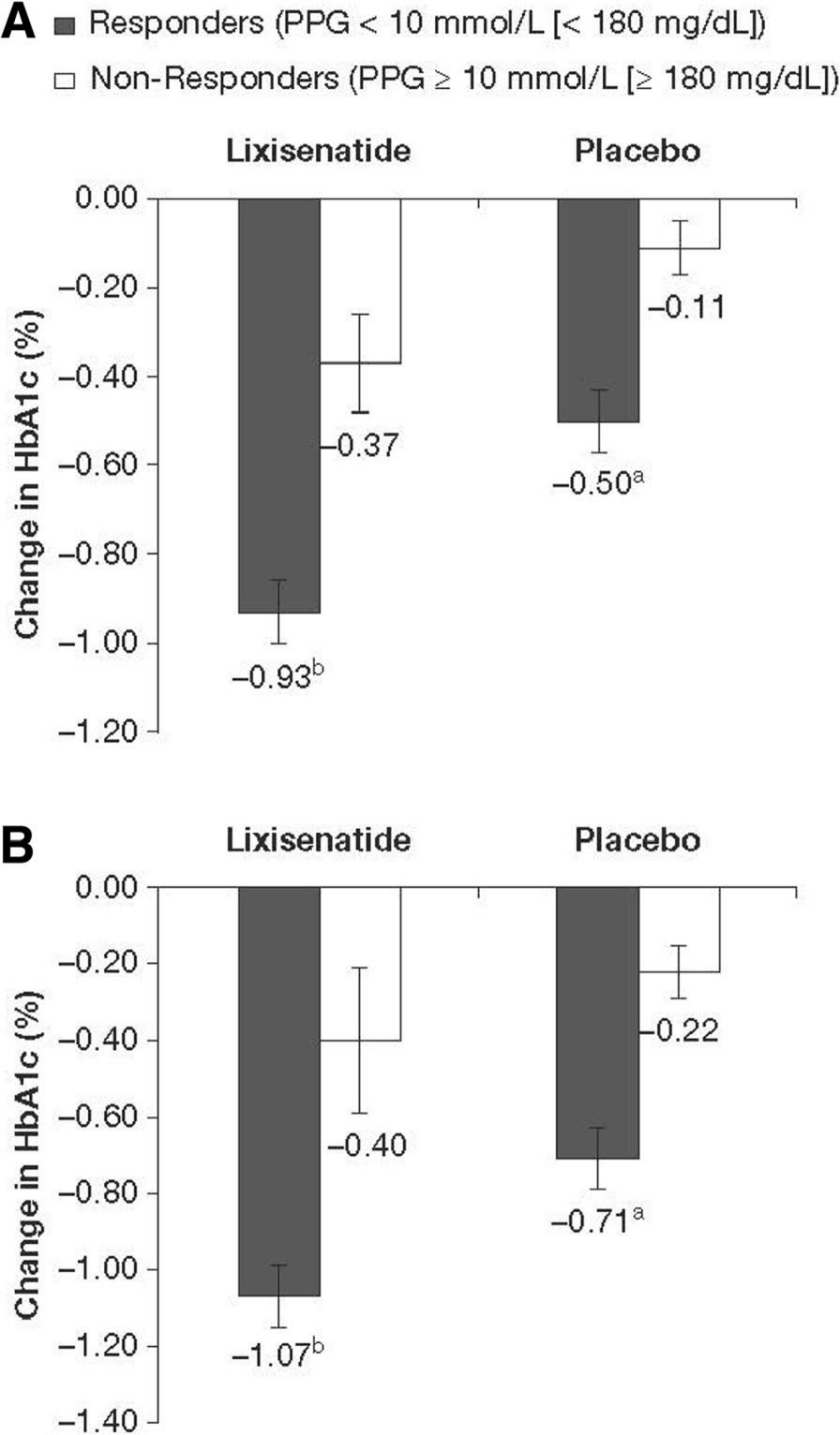
Change in HbA1c from baseline to Week 24. **a** Patients with controlled baseline FPG. **b** Patients with uncontrolled baseline FPG. *Abbreviations: HbA1c* glycated hemoglobin, *FPG* fasting plasma glucose, *PPG* postprandial glucose. ^a^*P* = 0.008. ^b^*P* < 0.001 for comparison between PPG response categories for both baseline FPG categories; Cochran–Mantel–Haenszel test stratified by study

**Fig. 3:**
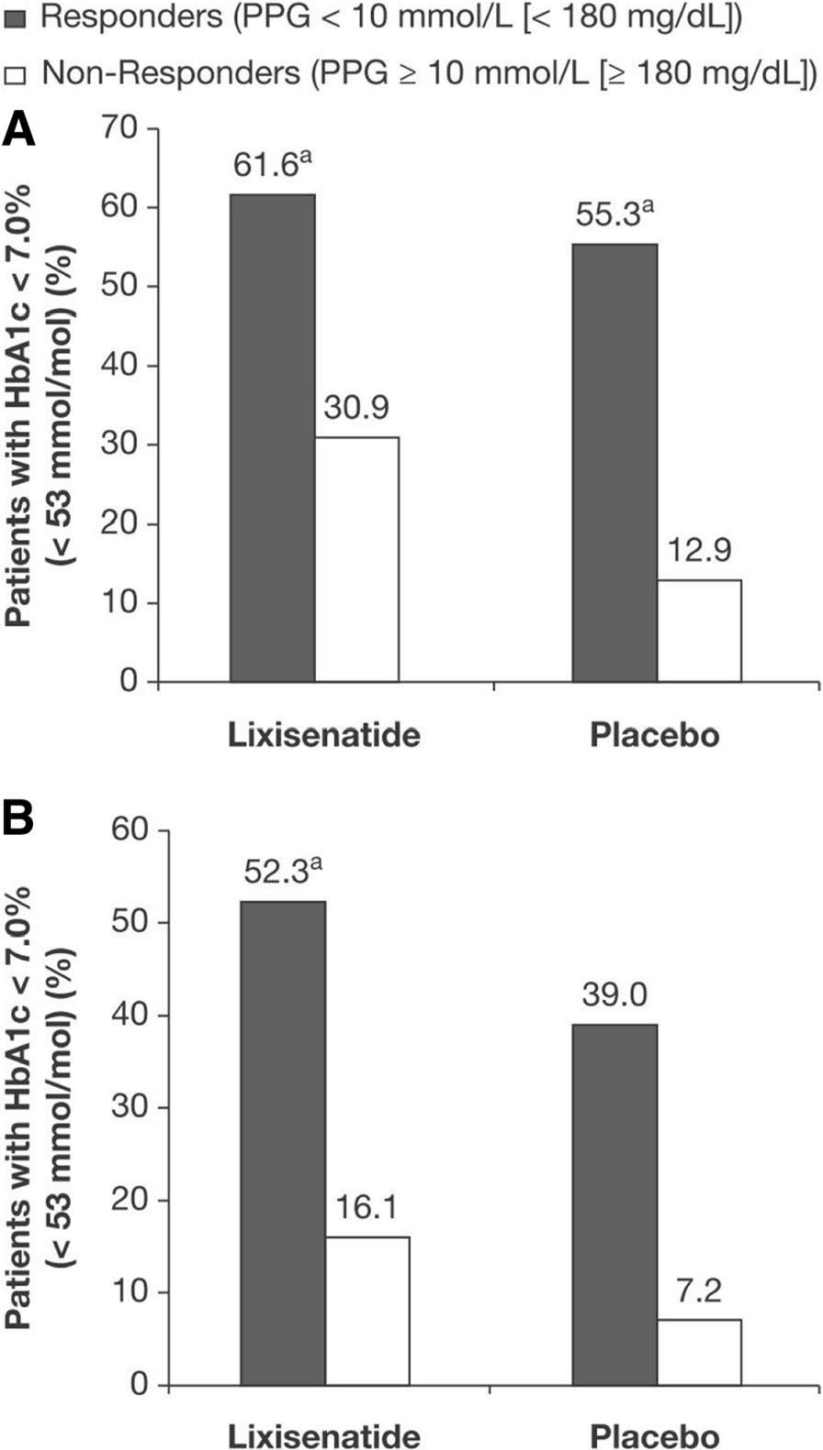
Percentage of patients with controlled (**a**) and uncontrolled (**b**) baseline FPG achieving HbA1c < 53 mmol/mol (< 7.0%) at Week 24. *Abbreviations: HbA1c* glycated hemoglobin, *FPG* fasting plasma glucose, *PPG* postprandial glucose. ^a^*P* < 0.001 for comparison between PPG response categories

Regardless of baseline FPG status, patients who reached PPG target achieved a significantly greater mean reduction in HbA1c compared with patients who did not reach PPG target. The magnitude of HbA1c change from baseline was similar for patients with controlled and uncontrolled FPG (Fig. 2; Table 2), with greater absolute HbA1c reductions in PPG responders than non-responders. Regardless of whether PPG target was achieved, patients with controlled FPG experienced increased FPG, whereas patients with uncontrolled FPG experienced FPG reductions. Among patients with controlled FPG, the increase was significantly lower in PPG responders, and among patients with uncontrolled FPG the reductions were significantly greater among PPG responders (Table 2). Patients who reached PPG target were also more likely to achieve HbA1c goal < 53 mmol/mol (< 7.0%) compared with patients who did not reach PPG target (Fig. 3).

**Table 2:** Summary of efficacy and safety outcomes in patients treated with lixisenatide

	Baseline FPG < 7 mmol/L (< 126 mg/dL) (controlled)			Baseline FPG ≥ 7 mmol/L (≥ 126 mg/dL) (uncontrolled)		
	PPG responders^a^ Lixisenatide (*n* = 156) Placebo (*n* = 47)	PPG non-responders^b^ Lixisenatide (*n* = 127) Placebo (*n* = 210)	Difference between groups (SE)	PPG responders^a^ Lixisenatide (*n* = 147) Placebo (*n* = 23)	PPG non-responders^b^ Lixisenatide (*n* = 157) Placebo (*n* = 204)	Difference between groups (SE)
Change in PPG from baseline to Week 24, mean (SE), mmol/L						
Lixisenatide	−7.75 (0.3)	−1.09 (0.4)	−6.69 (0.5)^c^	−9.20 (0.4)	−2.50 (0.4)	− 6.37 (0.5)^c^
Placebo	−3.55 (0.6)	0.84 (0.3)	−5.32 (0.7)^c^	−6.13 (0.9)	−0.26 (0.3)	−6.22 (1.0)^c^
Change in FPG from baseline to Week 24, mean (SE), mmol/L						
Lixisenatide	0.54 (0.2)	1.63 (0.2)	−1.05 (0.2)^c^	−1.83 (0.2)	−0.51 (0.2)	−1.43 (0.3)^c^
Placebo	0.11 (0.3)	1.39 (0.1)	−1.16 (0.3)^c^	−3.12 (10.6)	−0.88 (0.2)	−2.05 (0.6)^c^
Patients with HbA1c goal < 53 mmol/mol (< 7.0%) at Week 24, %						
Lixisenatide	61.6	30.9	−26.7 (5.6)^c^	52.3	16.1	−32.7 (5.0)^c^
Placebo	55.3	12.9	−27.2 (8.2)^c^	39.0	7.2	−17.3 (10.7)
Change in HbA1c from baseline to Week 24, mean (SE), mmol/mol						
Lixisenatide	−10.1 (0.71)	−4.1 (0.80)	−6.1 (1.07)^c^	−11.7 (0.90)	−4.4 (0.87)	−7.1 (1.26)^c^
Placebo	−5.4 (1.21)	−1.2 (0.61)	−3.7 (1.40)^d^	−7.7 (2.12)	−2.4 (0.76)	−4.4 (2.30)^d^
Symptomatic hypoglycemia during 24-week study period, rate (SE)						
Lixisenatide	0.40 (0.04)	0.36 (0.04)	0.03 (0.06)	0.26 (0.04)	0.19 (0.03)	0.06 (0.05)
Placebo	0.13 (0.05)	0.23 (0.03)	−0.05 (0.06)	0.12 (0.07)	0.16 (0.03)	−0.04 (0.08)
Body weight change from baseline to Week 24, mean (SE), kg						
Lixisenatide	−1.22 (0.23)	−0.10 (0.25)	−1.23 (0.34)^c^	−0.73 (0.19)	−0.27 (0.19)	−0.62 (0.28)^e^
Placebo	0.99 (0.41)	0.55 (0.19)	0.09 (0.47)	0.05 (0.54)	0.01 (0.16)	−0.05 (0.57)
Change in insulin dose from baseline to Week 24, mean (SE), mmol/L						
Lixisenatide	−0.61 (0.50)	−2.44 (0.61)	1.36 (1.18)	−0.37 (0.60)	0.24 (0.54)	−0.39 (1.23)
Placebo	1.80 (0.79)	−0.16 (0.28)	1.87 (1.12)	1.26 (0.96)	1.25 (0.44)	−0.24 (1.72)

Data were derived from a fixed-effects meta-analysis with inverse variance weights. Changes in HbA1c, PPG, FPG, body weight and insulin were analyzed using last observation carried forward at Week 24

*Abbreviations: HbA1c* glycated hemoglobin, *FPG* fasting plasma glucose, *PPG* postprandial glucose, *SE* standard error

^a^PPG < 10 mmol/L (< 180 mg/dL). ^b^PPG ≥ 10 mmol/L (≥ 180 mg/dL). ^c^*P* ≤ 0.001. ^d^*P* = 0.008. ^e^*P* = 0.024

There was no significant change in the risk of symptomatic hypoglycemia associated with achieving PPG target (Table 2). Patients who reached PPG target also experienced greater average reductions in body weight compared with those who did not reach PPG target with lixisenatide treatment; in contrast, all the placebo groups showed an increase in body weight (Table 2).

## Discussion

The results of this post hoc, pooled analysis of patient data from the GetGoal-L, GetGoal-Duo 1, and GetGoal-L Asia trials found that adding the short-acting GLP-1 RA lixisenatide to basal insulin in patients with T2D improves control of postprandial hyperglycemia, with more than half of patients achieving the ADA-recommended PPG target of < 10 mmol/L (< 180 mg/dL). Patients whose PPG reached target through treatment with lixisenatide were also more likely to achieve ADA-recommended HbA1c target of < 53 mmol/mol (< 7.0%) than patients who continued on basal insulin plus placebo. This was true regardless of whether or not patients had an FPG <  7 mmol/L (< 126 mg/dL) at baseline, but the observed effect was greater in patients with controlled FPG compared with patients with uncontrolled FPG.

These results are consistent with those of studies investigating other GLP-1 RAs combined with basal insulin. When exenatide, another GLP-1 RA, was added to insulin glargine, patients with T2D experienced a − 1.74% change in HbA1c which was completely driven by reductions in PPG [19]. This is further supported by a trial comparing exenatide and lispro with optimized basal insulin, which showed the non-inferiority of exenatide, with a change of − 1.13% (12.4 mmol/mol) in HbA1c [20].

In this analysis, the greater efficacy of lixisenatide compared with placebo in improving glycemic control was achieved without any increased risk of hypoglycemia and with body weight reduction, especially in patients achieving the PPG target. In a previous study, continuous glucose monitoring of patients administered lixisenatide at breakfast or the main meal of the day demonstrated reduced glucose exposure and a reduction of HbA1c of 0.6% over the 24-week study period [21]. This greater efficacy, together with mitigation of unwanted side effects, such as body weight gain, without increasing the risk of hypoglycemia, may aid in removing obstacles to treatment intensification.

## Conclusion

In this pooled analysis of the three GetGoal trials, addition of lixisenatide to basal insulin improved HbA1c and reduced postprandial glucose, regardless of baseline fasting plasma glucose, with no increased risk of symptomatic hypoglycemia while mitigating weight gain in patients with T2D. These findings support the hypothesis that achieving PPG < 10 mmol/L (< 180 mg/dL) with lixisenatide increases the likelihood of HbA1c goal achievement in patients with T2D, and further highlights the importance of PPG control in diabetes management.

## Data Availability

Please contact author for data requests.

## Abbreviations

AACE: American Association of Clinical Endocrinologists
ACE: American College of Endocrinology
ADA: American Diabetes Association
BMI: Body mass index
EASD: European Association for the Study of Diabetes
FPG: Fasting plasma glucose
GLP-1: Glucagon-like peptide-1
HbA1c: Glycated hemoglobin;
IDF: International Diabetes Federation
MET: Metformin
OAD: Oral antidiabetes drug
PPG: Postprandial glucose
RA: Receptor agonist
RAI: Rapid-acting insulin
SD: Standard deviation
SE: Standard error
SU: Sulfonylurea
T2D: Type 2 diabetes
TZD: Thiazolidinediones
